# Community networks of services for pregnant and parenting women with problematic substance use

**DOI:** 10.1371/journal.pone.0206671

**Published:** 2018-11-19

**Authors:** Karen Urbanoski, Chantele Joordens, Gillian Kolla, Karen Milligan

**Affiliations:** 1 Canadian Institute for Substance Use Research, University of Victoria, Victoria, British Columbia, Canada; 2 Centre for Addiction and Mental Health, Toronto, Ontario, Canada; 3 Dalla Lana School of Public Health, University of Toronto, Toronto, Ontario, Canada; 4 Department of Psychology, Ryerson University, Toronto, Ontario, Canada; Erasmus Medical Center, NETHERLANDS

## Abstract

Integrated treatment programs for pregnant and parenting women who use substances operate at the intersection of multiple service systems, including specialized substance use services, the broader health system, child protection, and social services. Our objectives were to describe the composition and structure of community care networks surrounding integrated treatment programs in selected communities in Ontario, Canada. We used a two-stage snowball method to collect network data from 5 purposively selected integrated treatment programs in communities in Ontario. Front-line staff with integrated treatment programs identified their top 5 service partners, who were then contacted and asked to provide the same information (n = 30). We used social network analysis to measure the cohesiveness, reciprocity, and betweenness centrality in the integrated treatment program’s ego network. We described network composition in terms of representation of different service types. Across communities, common service partners were child protection, substance use or mental health services, parenting and child support, and other social services. Primary and pre-natal care, opioid agonist therapy, and legal services were rarely named as partners. Networks varied in network cohesiveness, as indicated by connectivity between the service partners and reciprocal ties to the integrated treatment programs. Integrated treatment programs commonly brokered the connections between other service partners. Findings suggest that these integrated treatment programs have achieved a level of success in developing cross-sectoral partnerships, with child protection services, parenting and child support, and social services featuring prominently in the networks. In contrast, there was a lack of close connections with physician-based services, highlighting a potential target for future quality improvement initiatives in this sector.

## Introduction

Women who are experiencing problems related to their use of drugs and alcohol are typically of child-bearing age, and many are balancing responsibilities of motherhood [1, 2]. It is widely recommended that substance use services for women attend to their unique needs and contexts, and reduce barriers that make it difficult for women to participate in substance use services (e.g., lack of childcare, fear of loss of child custody, limited services for pregnant women, provider stigma) [3–11]. Integrated treatment programs have been developed that cater to women who are pregnant or parenting, using case management and service partnerships to overcome the traditional fragmentation of service sectors and offer a comprehensive array of health and social services. Meta-analyses have reported that integrated treatment helps women to reduce their substance use, and is associated with superior maternal mental health, birth and child health outcomes relative to non-integrated treatment [12–15].

There is no single standard definition or service model for integrated treatment programs for pregnant and parenting women, and no accepted policy or funding standards that dictate a minimum number or type of services or partnerships. In practice, programs that claim to offer integrated treatment contain a heterogeneous mix of services depending on local needs and resources [16]. There is a lack of research on the implementation and delivery of services within integrated treatment programs [17], and so we know little about the types of service partnerships that programs develop on the ground as they build community care networks for service provision. In this study, we use a network approach to investigate the partnerships between integrated treatment programs and ancillary health and social services, using measures of network composition and structure to contribute to better understandings of the heterogeneity in integrated service delivery across communities.

At a conceptual level, integrated treatment programs are guided by a set of core principles and practices, including: care that is holistic, empowering, and tailored to women’s needs; strong investment in staff and organizational health; innovative and coordinated partnerships; and supportive policies from multiple service sectors [18]. Specific service complements vary but commonly include specialized substance use services, maternal and child mental health care, pre-natal and primary care, child protection services, parenting programs, child-minding, and supports for social determinants of health (e.g., housing, income supports, transportation assistance) [3, 11, 16, 19]. Programs typically rely on strong collaborative arrangements for service delivery, sometimes with services co-located under a single roof (i.e., a one-stop shop model), with others relying to a greater or lesser extent on formal and informal partnerships with agencies in their communities (i.e., distributed service model). The key idea across service models is that by working together through cross-sectoral and collaborative networks, service providers are better able to meet the complex needs of women and their children [3, 20].

Given the strong role that partnerships play in service delivery, there is value in investigating how the community care networks that underlie integrated treatment programs are structured. Individual services and care providers are embedded within larger health systems, and there is increasing recognition of the utility of using the concepts and methods of social networks research to evaluate this embeddedness and its meaning for service delivery and outcomes [21, 22]. Social network analysis offers a set of methods to map and measure the relationships between actors (e.g., people, organizations, service providers) [23, 24]. Applied to the study of interdisciplinary health coalitions, collaboratives, or care networks, social network analysis can be used to examine the interconnections between service providers or organizations, cross-sectoral partnerships, diversity in network membership, and the existence of silos (i.e., networks or subcomponents of networks comprised of providers of a single discipline or type) [25–27]. Findings can provide insights into the development and sustainability of health networks, and linked to downstream measures of network efficiency, productiveness, and impacts [28].

In essence, integrated treatment programs for pregnant and parenting women are envisioned to be the focal service in community care networks comprised of an array of health and social services.They may serve as a hub, hosting services of different types, or they may provide an important coordinating function, bringing different service providers to facilitate care. Beyond providing a tally of services offered within or as part of an integrated program, evaluations of network structure provide a way to examine this focal role and model the connectedness of services belonging to care networks, including partnerships within and across sectors or disciplines. Programs that are connected to a wider variety of service types, either directly or indirectly, may be better able to access resources and supports for their clients (i.e., akin to having greater social capital) [23]. An understanding of these connections between providers within and across service sectors may help to illustrate the ways that clients move through the system, pinpoint potential gaps and points of weakness in community care networks, and inform strategies for improving care quality. No prior studies have evaluated the structures of community care networks for integrated service provision for pregnant and parenting women with problematic substance use.

### Evaluation context

In the province of Ontario (est. 2015 population 13.8 million), a suite of integrated treatment programs for pregnant and parenting women who use substances has been operating since 2003. Housed in agencies that deliver specialized psychosocial treatment for problematic substance use, programs receive funding from the health ministry of the provincial government and are accessed free-of-charge by residents of Ontario. Programs were developed to meet local needs, and evaluations conducted early on suggest that they were successfully meeting the needs of women and their children [29, 30]. Partnerships and services have continued to evolve the past decade to accommodate changing needs and local resources. A systematic evaluation of the community care networks created to support service delivery within these programs has never been conducted.

### Objectives

As part of a comprehensive evaluation of treatment processes and outcomes of integrated programs, we sought to describe the composition and structure of partnership networks in different communities. Our objectives were to: 1) describe the types of services that partner with integrated treatment programs to meet the needs of pregnant and parenting women and their children; and 2) evaluate the cohesiveness of these community care networks and the structural position of integrated treatment programs within them (i.e., their focal role). A secondary focus was on describing the differences in network composition and structure across communities.

## Materials and methods

This study is part of a larger mixed methods evaluation of a suite of 34 integrated treatment programs in Ontario, Canada. For this study, we purposively selected five integrated treatment programs to obtain variability in program size and characteristics (e.g., one-stop-shop vs. distributed services), and geography. All five integrated treatment programs had been admitting clients since at least 2003. A brief description of selected programs is provided in Table 1, based on information collected during site visits and interviews with program staff and leadership; city-size designations are defined using OECD definition of urban populations [31]. The descriptions are anonymized to protect agency confidentiality. Data were collected from the integrated treatment programs between June and November 2015, and from their service partners between February and May 2016.

**Table 1:** Characteristics of purposively selected integrated treatment programs in Ontario, 2016.

A	B	C	D	E
Serves approx. 100 women annually (<12 years old). Strives to provide a one-stop shop for clients, with a variety of services offered onsite. Located in a medium-sized urban area (pop. 200,000 to 500,000).	Serves more than 200 women annually (<16 years old). Strives to provide a one-stop shop for clients, with a variety of services offered onsite. Located in a large metropolitan area (pop. > 1.5 million).	Embedded within a treatment agency for youth. Serves approx. 30 young women annually (<25 years old). Follows a distributed model of care, with services offered primarily through partnerships with external agencies. Located in a metropolitan area (pop. 500,000 to 1.5 million)	Smaller sized program that expanded over time to serve more than 200 women (<16 years old) annually. Follows a mixed model, with a variety of services offered onsite and through partnerships with external agencies. Located in a medium-sized urban area (pop 200,000 to 500,000).	Serves approx. 40 women annually. Follows a mixed model, with a variety of services offered onsite and through partnerships with external agencies. Located in a small urban area (pop. 50,000 to 200,000).

### Participant recruitment and data collection

Network data were collected using a two-stage snowball method [32]. During site visits, we conducted face-to-face structured interviews with a front-line counselor working with the program. One point-person per program was interviewed for this study. The only selection criteria were that the participant be closely acquainted with and personally involved in service delivery in the integrated treatment program. We asked participants to nominate and provide contact information for their top five service partners. “Partnership” was defined as “*service agencies in your community to which you refer and accept clients*, *agencies with which you have service and/or data sharing agreements*, *and/or services that you typically help your clients access*.*”* We subsequently contacted the nominated partners by telephone and asked them to nominate their top five service partners, using the same question. The partner participants were not aware of each other’s identity at the time of the interview, although they were aware of the identity of the integrated treatment program and that they had been named as a top service partner by that agency. We conducted 30 interviews in total (6 per community, including the program plus the 5 service partners). The response rate among service partners was 100%. All participants provided written informed consent.

The resulting data were used to construct five ego networks comprised of nodes (service providers) and the connections or ties between them defined by partnership nominations. The networks include an *ego* (i.e., the focal node, in this case, the integrated treatment program), *1-step alters* (i.e., service partners directly connected to the ego/integrated treatment program) and *2-step alters* (i.e., service partners indirectly connected to the ego through a 1-step alter). Ties between agencies are binary (present/absent) and have direction (they start at one service provider and lead to another). The 1-step alters could, and often did, nominate other 1-step alters in their top five service partners (i.e., indicating duplication in the top five service partners named by the ego and 1-step alters). Ties between 2-step alters were not collected.

Participants were not directly compensated for their time. Participating integrated treatment programs received, as part of the larger evaluation, a $100 gift certificate to a books/home goods store chain, to purchase toys and books for their program. Study activities were approved by the Institutional Review Boards at the Centre for Addiction and Mental Health and Ryerson University, in Toronto, Ontario.

### Measures

Service partners (i.e., 1-step and 2-step alters) were grouped into nine service types. *Other substance use or mental health services* included psychologists, psychiatrists, and all counseling services with a primary mandate to address substance use or mental health, excluding opioid agonist therapy (OAT; i.e., buprenorphine/naloxone or methadone). *OAT* providers and clinics were grouped in their own category. *Prenatal services* included prenatal classes or groups, maternity and birthing centers, and obstetricians. *General medical and primary care* included family doctors, general practitioners, pediatricians, nurses, and nurse practitioners, as well as general hospitals, health clinics, and community health centers. *Parenting and child support services* included developmental assessment for children, childcare or babysitting, child playgroups, and parenting education or support. *Child protection services* represent government-based and non-profit agencies with a primary mandate for child protection. In Ontario, there are a variety of child protection agencies, with agencies specific to religion and with agencies specific to Indigenous communities. *Legal services* included legal aid and counsel. *Public health services* included regional and municipal public health units. *Social services* included services with a primary mandate for advocacy, housing support, shelters, respite care, domestic violence, and general help-lines. This typology was defined by the research team, guided by program descriptions generated using data from interviews with program staff and by prior research in this area [20].

We calculated three measures of the structure of the ego networks, which draw attention to the connections between the service partners. Consistent with ego network analysis (described below), only the services that were directly nominated by the integrated treatment program (1-step alters) participated in the calculation of these measures. *Effective size* is a summary measure of network structure based on the ties between 1-step alters [32]. In this case, it represents the extent to which the services that were nominated by the integrated treatment program are themselves connected to one another (i.e., they were on *each other’s* respective lists of top five service partners). Effective size is calculated as the number of 1-step alters (which in this study is fixed at 5) minus the average number of ties per alter. Values close to 5 indicate a low level of connections between the service partners; smaller values indicate increasing levels of connectedness between the service partners (i.e., higher network cohesiveness). We also calculated ego *in-degree*, or the number of times that the ego was nominated by a 1-step alter. This provides a measure of reciprocity of the ties between the integrated treatment program and its service partners. By design, all of the service partners (1-step alters) were nominated by the ego (integrated treatment program); ego in-degree provides a count of the service partners who reciprocated by naming the integrated treatment program as one of their top five partners. We report in-degree as both the number of and proportion of reciprocated partner ties. Finally, we calculated ego *betweenness centrality*, or the extent to which the ego lies on the shortest path between two 1-step alters [33]. Betweenness centrality is a measure of brokerage, or the extent to which the integrated treatment program (ego) is a point of connection between other services (1-step alters) that are themselves not connected to one another [22]. Integrated treatment programs with high betweenness are therefore acting as a bridge between services in their care network; they are bringing new expertise and knowledge into the network. Within networks, high betweenness signifies greater prominence and advantages in terms of access to unique and diverse perspectives and resources (i.e., social capital) [22, 34].

### Data analysis

The five community care networks were analyzed separately. The analysis focused on the integrated treatment program (ego), as a service provider embedded within a larger network of service providers of different kinds (1-step and 2-step alters). We created sociograms to visually display network connectivity, with spring embedding to determine where nodes are positioned in the diagram. Spring embedding uses an iterative algorithm to arrange the nodes in the diagram, such that nodes separated by smaller distances (e.g., directly connected to one another) are placed closer to one another in 2-dimensional space [32]. When determined using this approach, the positions of the nodes and the distances between them are meaningful (as opposed to a diagram in which node placement is determined randomly).

To evaluate the composition of these networks, we tabulated the types of partners represented in the ego and 2-step networks. Within networks, we tallied the number of partner types to provide an indication of diversity in service complement that is accessible through the integrated treatment programs. Combining across networks, we then tallied the number of partners of each type to provide an indication of the level of representation of services of different kinds.

We then used social network analysis to calculate the three measures of the shape and structure of the ego-networks (effective size, in-degree, and betweenness centrality) [23]. The ego network was extracted for quantitative analysis (i.e., the network corresponding to the ego and 1-step alters, and the ties between them). Results were examined by integrated program size (i.e., annual client volume) and city-size designation, reported in Table 1. The social network analysis was conducted in UCINET 6.598 [35] and Netdraw 2.157 [36] to create the sociograms.

## Results

**Fig 1:**
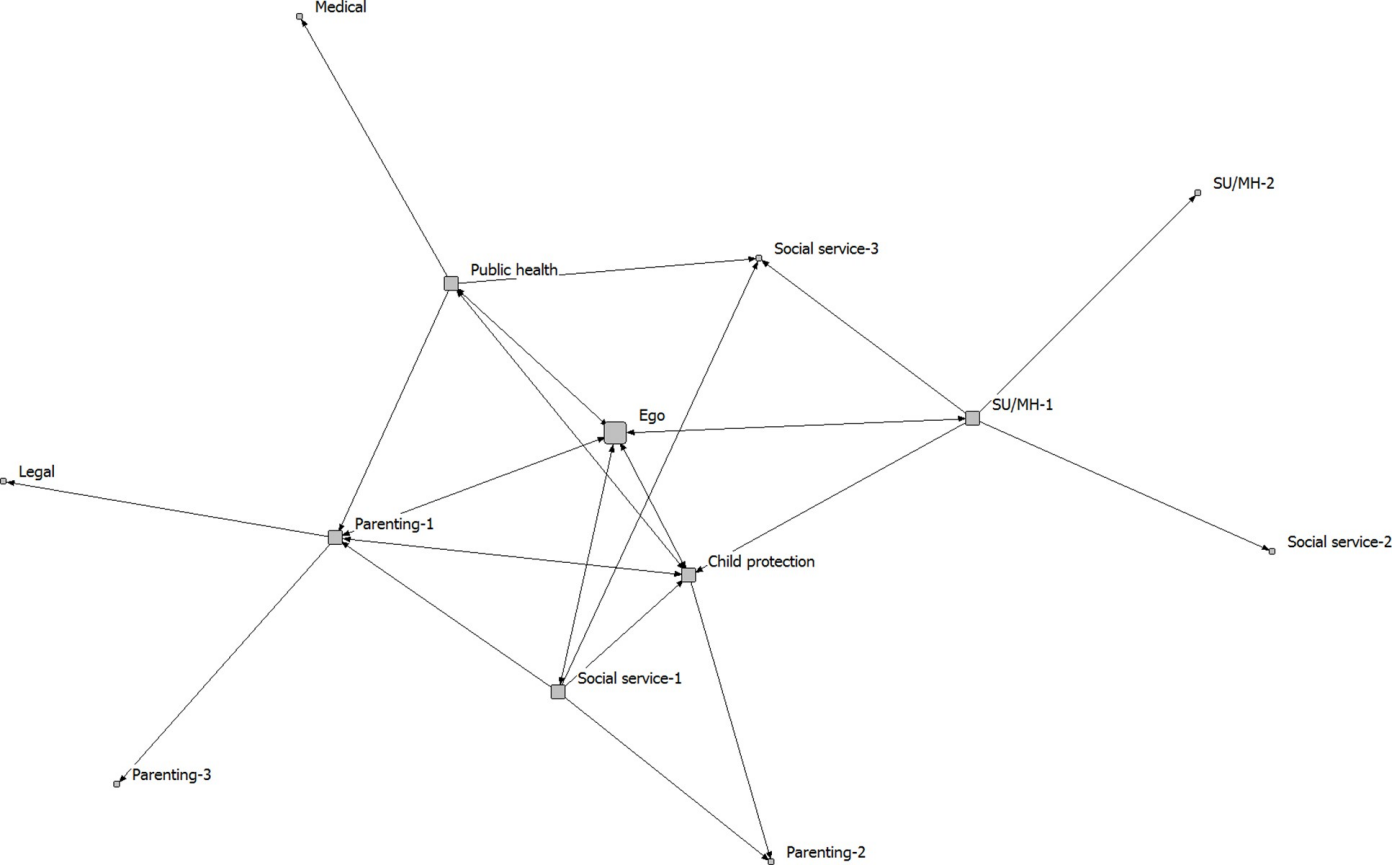
Network A.

**Fig 2:**
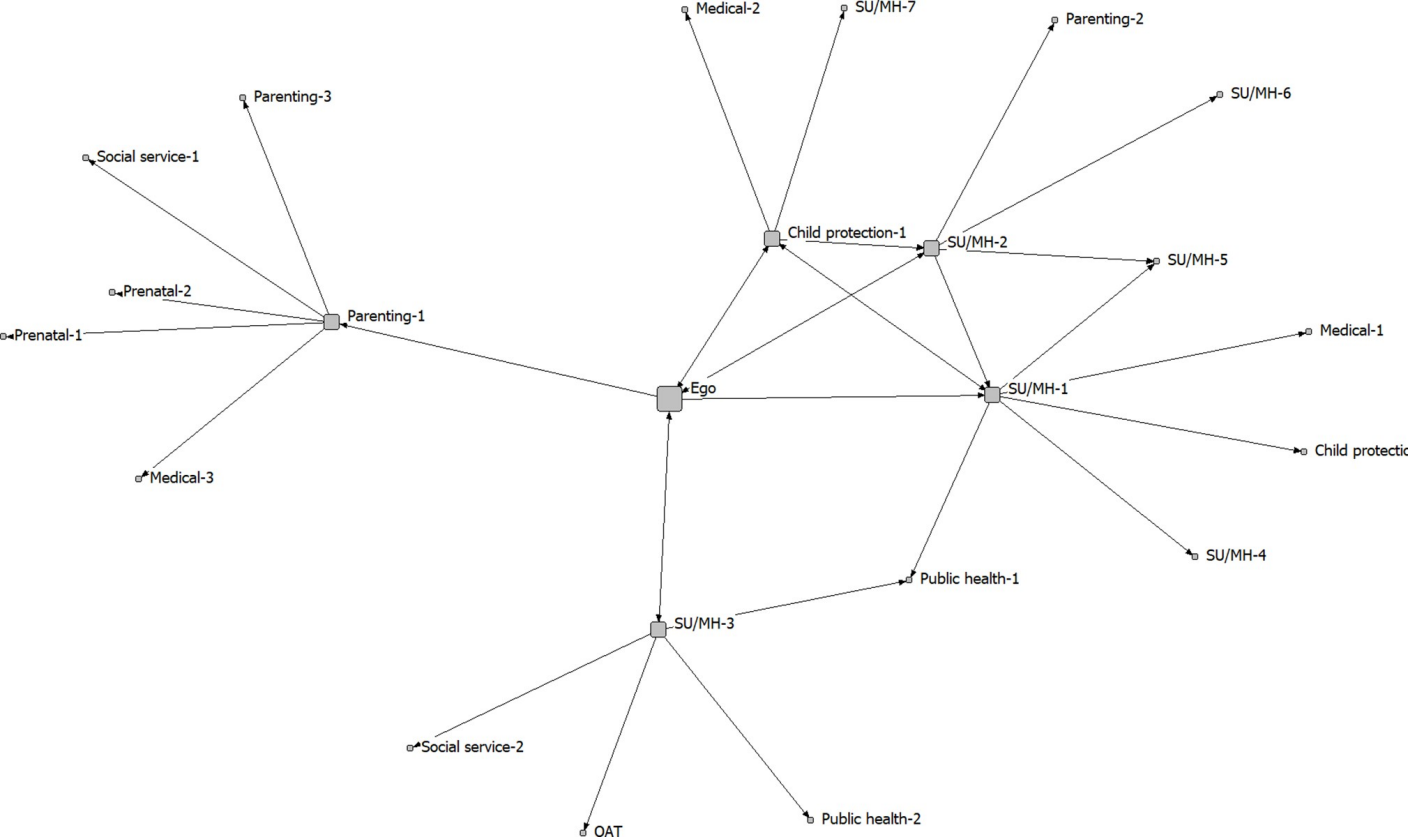
Network B.

**Fig 3:**
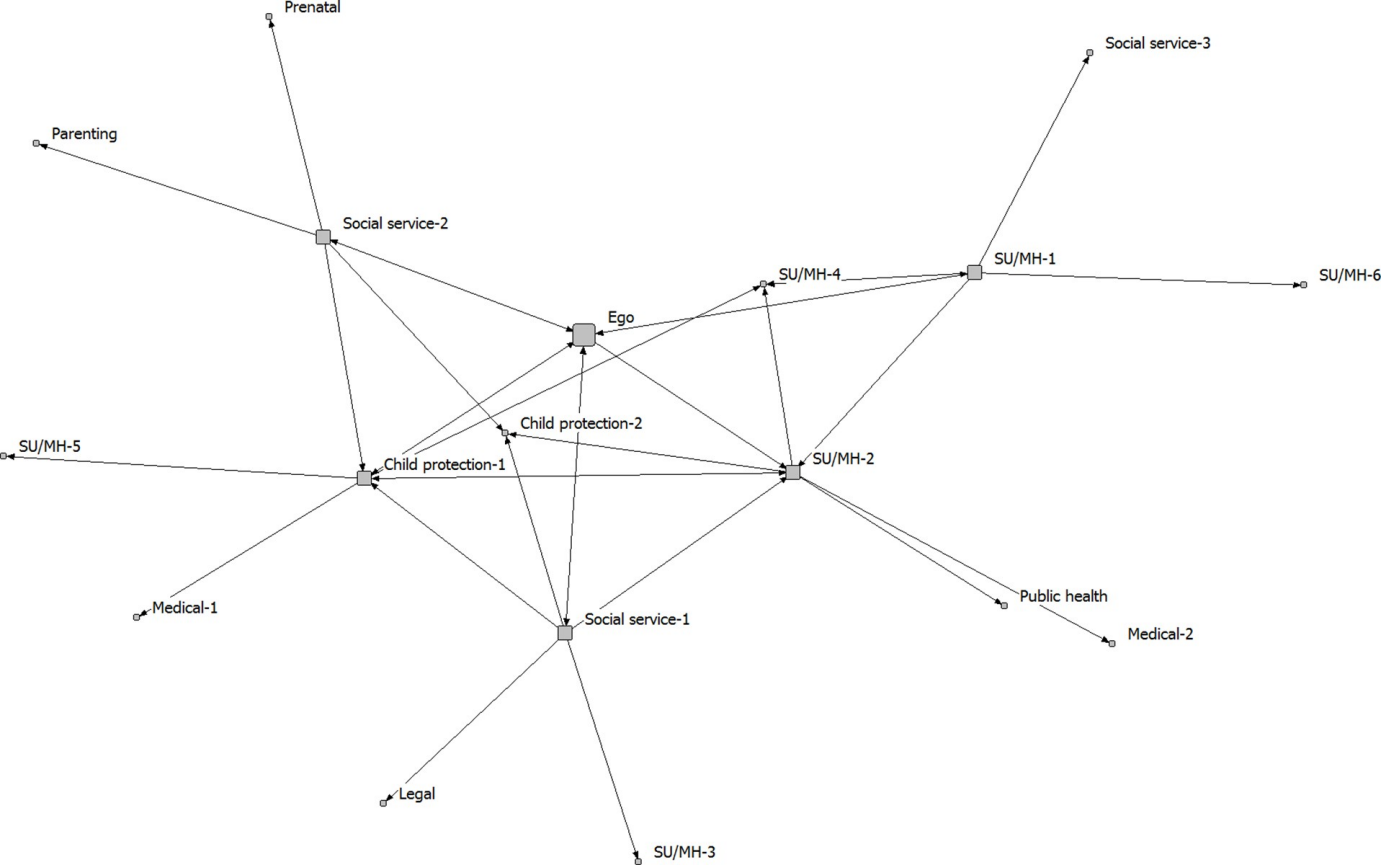
Network C.

**Fig 4:**
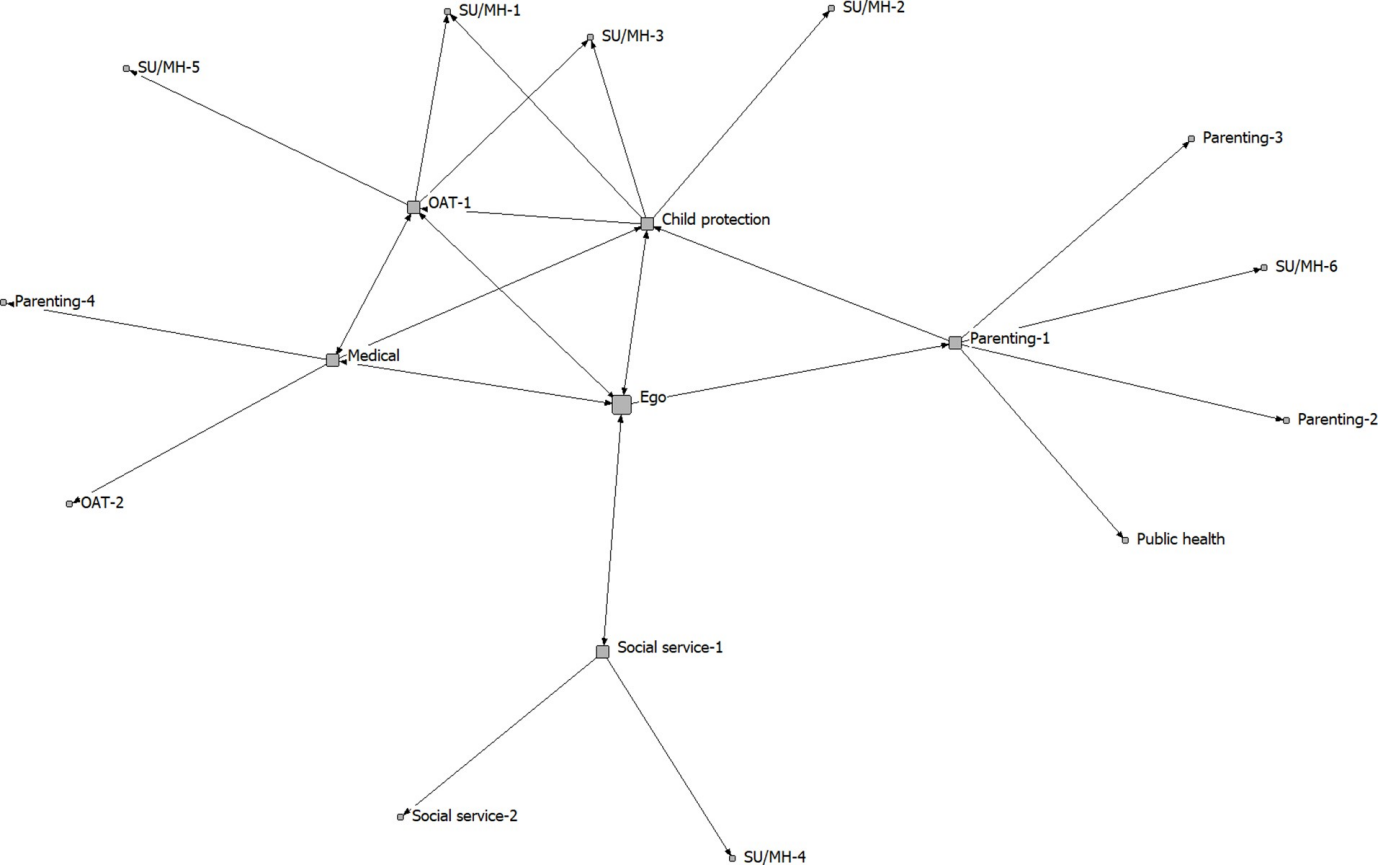
Network D.

**Fig 5:**
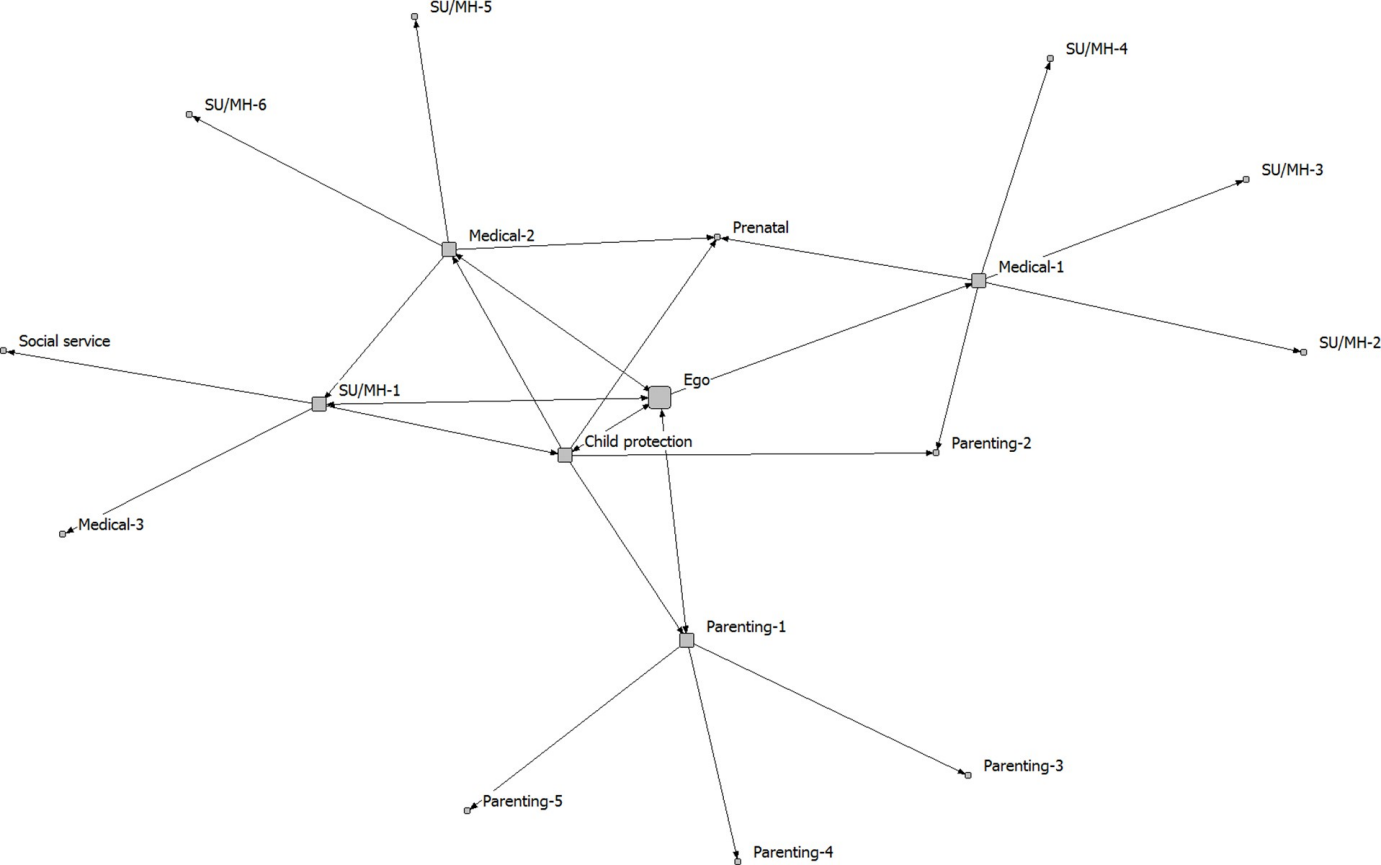
Network E.

The full networks, including both 1-step and 2-step alters, are shown in Figs 1–5. Service partners are labeled by type, with node size differentiating between 1-step (larger) and 2-step (smaller) alters. The connections between services are directed, with 2-headed arrows indicating a reciprocal relationship (the services named each other as key partners). These sociograms provide a visual representation of the variability in density and direction of connections across these community care networks.

Tables 2–4 summarize the composition of service partner types in the community care networks. Partners comprised a range of service types, with some variability across communities. Considering just the 1-step alters (i.e., service partners directly connected to the integrated treatment program), child protection was the most commonly named service partner: all of the community care networks contained child protection services as a 1-step alter (Table 2). Most of the networks also had at least one other substance use or mental health service (exclusive of OAT), parenting or child support service, and social services. In contrast, only two of the ego networks contained medical/primary care services or public health, one contained an OAT provider, and none contained prenatal or legal services. Out of a potential maximum of 5, the number of distinct partner types represented in the care networks varied from 3 to 5 (Table 4).

**Table 2:** Summary of service types represented in the ego networks of selected integrated treatment programs in Ontario, 2016 ^a^.

A	B	C	D	E
Substance use/mental health (SU-MH)	Substance use/mental health (SU-MH) (3)	Substance use/mental health (SU-MH) (2)		Substance use/mental health (SU-MH)
			OAT	
			Medical or primary care	Medical or primary care (2)
Parenting/child support	Parenting/child support		Parenting/child support	Parenting/child support
Child protection	Child protection	Child protection	Child protection	Child protection
Public health				
Social services		Social services (2)	Social services	

^a^ Ego network includes service partners that are directly tied to the integrated treatment program (1-step alters); prenatal and legal services were not represented in any of these networks; where multiple service types of one kind are represented, the number is indicated in brackets

**Table 3:** Summary of service types represented in the 2-step networks of selected integrated treatment programs in Ontario, 2016 ^a^.

A	B	C	D	E
Substance use/mental health (SU-MH) (2)	Substance use/mental health (SU-MH) (7)	Substance use/mental health (SU-MH) (6)	Substance use/mental health (SU-MH) (6)	Substance use/mental health (SU-MH) (6)
	OAT		OAT (2)	
	Prenatal (2)	Prenatal		Prenatal
Medical or primary care	Medical or primary care (3)	Medical or primary care (2)	Medical or primary care	Medical or primary care (3)
Parenting/child support (3)	Parenting/child support (3)	Parenting/child support	Parenting/child support (4)	Parenting/child support (5)
Child protection	Child protection (2)	Child protection (2)	Child protection	Child protection
Legal services		Legal services		
Public health	Public health (2)	Public health	Public health	
Social services (3)	Social services (2)	Social services (3)	Social services (2)	Social services

^a^ 2-step network includes service partners that are directly tied to the integrated treatment program (1-step alters) and those indirectly tied to the integrated treatment program (2-step alters); where multiple service types of one kind are represented, the number is indicated in brackets

**Table 4:** Size and diversity in networks of selected integrated treatment programs in Ontario, 2016^a^.

	A		B		C		D		E	
	Ego	2-step	Ego	2-step	Ego	2-step	Ego	2-step	Ego	2-step
Total number of partners	5	12	5	22	5	12	5	17	5	17
Number of partner types	5	7	3	8	3	8	5	7	4	6

^a^ Ego network includes ego and 1-step alters and the ties between them; 2-step network also includes the 2-step alters

Expanding the network to include the 2-step alters (those not directly connected to the integrated treatment program) had the effect of increasing the number and variety of services in the networks. Again, substance use/mental services, parenting/child support services, and social services were commonly identified as 2-step alters, while OAT and legal services were least common (Table 3). In two communities, a second child protection agency was named. Out of a potential maximum of 25 unique organizations, network size (i.e., the number of 1-step and 2-step alters) per network ranged from 12 to 22 (Table 4). The number of distinct partner types represented varied from 6 to 8 (out of a maximum of 9). Where there were multiple partners of a given type within a network, it was common for there to be multiple services offering substance use/mental health supports and, to a lesser degree, parenting/child supports.

Quantitative measures of structure of the ego networks (i.e., the network corresponding to the ego and 1-step alters, and the ties between them) are shown in Table 5. Exclusive of the integrated treatment program, ego network size was fixed by design at 5, as each integrated treatment program was asked to nominate their 5 top service partners. However, the *effective size* of the ego networks varied from a low of 3.4 to 4.2, indicating variable levels of connectivity between the service partners. Across communities, the majority of connections between the 1-step alters and the ego were reciprocal, ranging from 60% (3/5 of the 1-step alters named the integrated treatment program as a key service partner in Community B) to 100% (5/5 of the 1-step alters reciprocated by naming the integrated treatment as a key service partner in Community A). Betweenness centrality ranged from 10 to 13, indicating that the integrated treatment programs occupied key positions in brokering connections between services within their community care networks, with little variation across communities.

**Table 5:** Structure of networks of selected integrated treatment programs in Ontario, 2016 ^a^.

	A	B	C	D	E
Effective size ^b^	3.4	4.2	3.9	3.8	4.1
In-degree ^c^	5	3	4	4	4
% of reciprocated ties ^d^	100%	60%	80%	80%	80%
Betweenness centrality ^e^	10	10.5	13	12.5	10

^a^ Ego network (n = 6 service providers); includes ego and 1-step alters, and the ties between them

^b^ Effective size = the extent to which the services that were nominated by the integrated treatment program are themselves connected to one another (i.e., they were on *each other’s* respective lists of top five service partners); calculated as the number of 1-step alters minus the average number of ties per alter

^c^ In-degree = number of times that the ego was nominated by a 1-step alter; providing a count of nominated service partners who reciprocated by naming the integrated treatment program as one of their top five partners

^d^ Calculated as in-degree/5

^e^ Betweenness centrality = how often the ego lies on the shortest path between two 1-step alters; providing an index of the extent to which the integrated treatment program (ego) is a point of connection between other services (1-step alters) that are themselves not connected to one another

Relative to the other communities, the network in Community A (a medium-sized city) was characterized by lower effective size and full reciprocity, meaning that the service partners all named the integrated treatment program as one of their key service partners and showed a fair amount of connectivity with each other independent of the integrated treatment program. This can be contrasted with the network in Community B (a large metropolitan area), which had higher effective size and lower reciprocity, meaning that there was less connectivity between the service partners and fewer reciprocal connections back to the integrated treatment program. In terms of the number of members, the network in Community A was the smallest of the 5 that we examined, while the network in Community B was the largest. These findings come through in the sociograms representing these two networks, with the greater density of ties between partners evident in Fig 1 (Community A) relative to Fig 2 (Community B). Notably, one of the parenting/child support services identified by the integrated treatment program in Community B identified five services that were not nominated by any of the other members of this community network (labeled as *Parenting-1* in Fig 2). In contrast, all of the 1-step alters had some degree of interconnectivity in Community A.

Although the range in betweenness centrality scores was not wide, the highest betweenness centrality was found for the integrated treatment program in Community D (a medium-sized city). This program was the only one of the 5 that was tailored specifically to youth (women <25 years old), although most of the other programs also included adolescents and young women within their clientele. In line with its relatively higher betweenness centrality, this program was also the only one of the 5 to describe itself explicitly as following a distributed service model (with services offered primarily through partnerships with external agencies).

There was no apparent pattern in the measures of network composition (number and types of partners) or structure (effective size, in-degree, reciprocity, or betweenness centrality) by the size of the program, defined as average numbers of women served per year (30–40 in Communities C and E; 100 in Community A; and 200 or more in Communities B and D).

## Discussion

The purpose of this study was to describe the composition and structure of community care networks involved in delivering services to pregnant and parenting women who use substances. Across communities, key partners were child protection services, as well as other substance use or mental health services, parenting and child support services (e.g., child developmental assessment, childcare, playgroups, parenting skills education or support) and social services (e.g., housing, domestic violence, respite care, advocacy). Other forms of health care (including OAT, primary care, and pre-natal care) and legal services were relatively rare in these community networks. There was variability across communities in network cohesiveness, as indicated by the extent to which partnerships were reciprocal and to which service partners were themselves inter-connected.

While there was some variability in the numbers and types of partners across community networks, findings highlight the prominence of child protection services, as well as services for substance use and mental health, parenting and child support, and social services. Prior research has demonstrated the importance of matching services to holistic needs for this population leads to longer retention in treatment and better substance use outcomes [37, 38]. The relative frequency of services for parenting, child support and protection, and social services (versus pre-natal, primary and medical care) is particularly interesting in this case as it indicates the successful development of cross-sectoral linkages (described as a core feature of effective integrated treatment by stakeholders in Ontario) [18]. These findings are notable, given the challenges associated with cross-sector partnerships. Particularly seen in substance use service provider and child protection collaborations, differing understanding of substance use and associated expectations and definitions of success complicate the process of working together. Further, resources (e.g., time, funding, leadership, education) are often limited yet required to establish the foundation for collaborative practice [38–40].

In these five community care networks, with the exception of substance use and mental health services, the services that featured most commonly represent different service sectors. That is, although they address broader social determinants of health, they are not health care services, per se. To some extent, this pattern of findings may reflect the primarily psychosocial, rather than medical, nature of substance use services within this system of care (for both the integrated treatment programs and the larger treatment agencies within which they are housed). Although specialized substance use services are funded and governed by the department of government focused on health (the Ministry of Health and Long-term Care in Ontario), they have traditionally functioned in practice quite separately from other forms of health care. The lack of close connections with physician-based services (e.g., OAT, psychiatry, primary care) signals the need for future development of partnerships and integration with local primary care services. In particular, the apparent lack of close connection to OAT prescribers is concerning. Given the current epidemic of overdoses related to opioids in Canada, and the fact that OAT is the first line of treatment for opioid use disorders [41], there is need for further study of the facilitators and barriers to implementing partnerships between OAT prescribers and services designed for pregnant and parenting women who use substances.

In a prior study, Sword et al. [20] also used a network approach to examine the connections between service agencies that provide services for women who use substances in Canada. Substance use treatment centres for women tended to partner with mental health services, with fewer referrals sent to social services, services for children (including child protection), or pre-natal care. Our findings suggest that integrated treatment programs are more likely to approach care in a holistic manner that includes partnering with services that provide support for parenting, child development, and social determinants of health. This is consistent with models of integrated care that highlight the importance of holistic care and attending to the needs of three clients, including mother, child and the mother-child relationship [18]. We also found, across all participating communities, that the integrated treatment programs occupied central positions within these community care networks, commonly brokering the connections between other service partners. This is similar to Sword et al. [20], who found large clusters of agencies centered around key substance use treatment providers in each province (agencies with high social capital and high potential for system enhancement and leadership). It should also be noted, however, that in all of the community networks that we evaluated, there were numerous other service providers who were also well-connected to other network members (i.e., had many ties to the other services who were part of the community care network; see Figs 1–5). These services may also play important roles within the networks, contributing to the overall effectiveness of the integrated service model in each community. Whole network approaches are required to capture the full scope of partnerships within these care networks [23].

There is no single optimal level of connectivity that could be expected to maximize the function of care networks across all contexts. In this case, from the perspective of the integrated treatment programs, having a set of service partners that are themselves connected to each other may bring advantages in terms of a shared client base and shared understandings of client needs, as well as each other’s mandates, philosophies, and therapeutic goals. The extent to which a high level of inter-connectivity results in less redundancy in assessment and treatment planning, for instance, or client perceptions of greater consistency in messaging and therapeutic goals, is an avenue for future research. Alternatively, it could also be that unconnected service partners bring benefits to the integrated treatment programs by offering resources or supports that are otherwise unavailable. Because the service partners directly connected to the integrated treatment programs (i.e., the 1-step alters) were asked to name their top five partners, lower connectivity between the 1-step alters means that new services were being identified or brought into the network as 2-step alters. It is possible that the integrated treatment programs benefit from these indirect connections with a wider array of service providers, who bring non-redundant or unique opportunities, different perspectives, or different ways of working. This diversity may be beneficial to service delivery to the extent that it allows the integrated treatment program to meet the needs of a more diverse array of clients. The same principle applies to betweenness centrality, in that network members that broker relationships between other members may benefit from occupying a position of strategic advantage within the network [22, 34]. Brokerage may bring opportunities for innovation and the transfer of knowledge and ideas, such that service providers with high betweenness centrality may be in a good position to shape the perspectives and practices of others in the care network. At the same time, occupying a brokerage role within a network can also come with costs in terms of work required to develop and maintain those ties [22], as noted above for collaborative practice between substance use and child protection services. Future work is needed to assess the costs and benefits to integrated treatment programs of playing a central role within the collaborative care networks needed to meet the needs of pregnant and parenting women who use substances.

Participants were limited to nominating 5 service partners. Although this strategy has the benefit of capturing the services that the agencies view as most important to care delivery for pregnant and parenting women who have problematic substance use, it is possible that other important service partners were missed. Further, we did not collect data on the top partners of 2-step alters. Absent ties and tie reciprocity must be interpreted with this limitation in mind. Further, we relied on the reports of a single point-person at each agency. While this may work reasonably well, particularly in smaller programs, it may have been less effective in larger agencies (i.e., those with a higher caseload). When there are a larger number of counsellors there may be more variability in who service providers partner with to meet the needs of their clients. Finally, we were unable to connect network characteristics with client-level outcomes, which would provide the most direct measure of their implications for program effectiveness.

### Conclusions

Integrated treatment programs play a prominent role within the community care networks for pregnant and parenting women who use substances. These care networks varied in levels of cohesiveness and reciprocity of partnerships, although there was fair degree of similarity across networks in the types of services that were represented. Findings suggest that these integrated treatment programs have achieved a level of success in developing cross-sectoral partnerships, with child protection services, parenting and child support, and social services featuring prominently in the networks. In contrast, there was a lack of close connections with physician-based services, highlighting a potential target for future quality improvement initiatives in this sector. Ultimately, further research is needed to elucidate the impact of these network features on program effectiveness, including the health and well-being of women and their children.
